# Multimodal Imaging from Fetal to Adult Life: A Comprehensive Approach to Hypoplastic Left Heart Syndrome (HLHS)

**DOI:** 10.3390/jcdd12090349

**Published:** 2025-09-11

**Authors:** Sara Moscatelli, Jolanda Sabatino, Isabella Leo, Nunzia Borrelli, Martina Avesani, Giovanni Di Salvo, Claudia Montanaro, Valeria Pergola, Raffaella Motta, Jessica Ielapi, Assunta Di Costanzo, Rosalba De Sarro, Giulia Guglielmi, Irene Cattapan, Gabriella Gaudieri, Leonie Luedke, Marco Alfonso Perrone

**Affiliations:** 1 Centre for Inherited Cardiovascular Diseases, Great Ormond Street Hospital, London WC1N 3JH, UKl.luedke@ucl.ac.uk (L.L.); 2 Institute of Cardiovascular Sciences, University College London, London WC1E 6BT, UK; 3 Paediatric Cardiology Department, Royal Brompton and Harefield Hospitals, Guy’s and St. Thomas’ NHS Foundation Trust, London SW3 5NP, UK; 4 Experimental and Clinical Medicine Department, University Magna Graecia of Catanzaro, 88100 Catanzaro, Italyassuntadicostanzo.med@gmail.com (A.D.C.);; 5 Adult Congenital Heart Disease Unit, A.O. dei Colli, Monaldi Hospital, 80131 Naples, Italy; 6 Division of Paediatric Cardiology, Department of Women and Children’s Health, University Hospital of Padua, 35128 Padua, Italy; 7 Unità Operativa Semplice di Cardiologia del Congenito Adulto, Bambino Gesù Children’s Hospital, IRCCS, 00165 Rome, Italy; 8 Dipartimento di Scienze Cardio-Toraco-Vascolari e Sanità pubblica, University Hospital of Padua, 35128 Padua, Italyraffaella.motta@unipd.it (R.M.); 9 Pediatric and Adult Congenital Heart Centre, IRCCS-Policlinico San Donato, Piazza Edmondo Malan, 2, 20097 Milan, Italy; 10 Clinical Pathways and Epidemiology Unit, Bambino Gesù Children’s Hospital IRCCS, 00165 Rome, Italy; 11 Division of Cardiology and Cardio Lab, Department of Clinical Science and Translational Medicine, University of Rome Tor Vergata, 00133 Rome, Italy

**Keywords:** hypoplastic left heart syndrome, cardiovascular imaging, congenital heart disease, pediatric cardiology

## Abstract

Hypoplastic Left Heart Syndrome (HLHS) accounts for 2–3% of congenital heart diseases (CHDs). HLHS is characterized by reduced systemic blood flow due to hypoplastic left ventricle (LV) and underdeveloped left-sided cardiac structures. Without a series of staged interventional treatments, HLHS is often fatal, typically within the first hours or days of life. This manuscript aims to provide a comprehensive overview of the role of echocardiography, cardiovascular magnetic resonance (CMR), and cardiac computed tomography angiography (CCTA) in the optimal management of patients with HLHS. Specifically, it explores the contributions of various non-invasive imaging modalities to the diagnosis, planning of staged palliative interventions, interstage monitoring, and long-term follow-up of HLHS. Furthermore, the advantages and limitations of each imaging technique will be highlighted to aid in clinical decision-making; however, it is important to note that, at present, no universal guidelines exist, and imaging strategies remain largely dependent on individual centre expertise and protocols.

## 1. Introduction

Hypoplastic Left Heart Syndrome (HLHS) is a rare but highly lethal congenital heart disease (CHD), responsible for 2–3% of all CHDs and 20–25% of CHDs-related deaths in the neonatal period [1,2,3]. The syndrome includes several cardiac anomalies, all sharing a hypoplastic left ventricle (LV) that is incapable of guaranteeing adequate systemic perfusion with variably associated hypoplasia of the ascending aorta, mitral or aortic valve stenosis [4,5]. In the first days of life, atrial septal defects (ASDs) and a patent ductus arteriosus (PDA) are critical for maintaining systemic circulation [6]. This is especially true in severe forms of HLHS, where aortic or mitral atresia prevents systemic circulation, unlike milder forms, where the hypoplastic LV may still partially contribute [6,7]. Without interventional treatment, HLHS is invariably fatal. However, survival can be achieved through a series of staged palliative interventions [8,9].

The primary goals for the first intervention, typically the Norwood procedure, are to ensure systemic blood flow, provide stable pulmonary blood flow for adequate oxygenation, and remove any restriction to pulmonary venous return [8,10]. This involves reconstructing the aorta using the native main pulmonary artery (MPA) along with the ascending aorta and arch, thereby allowing the right ventricle (RV) to support systemic circulation [10,11]. A shunt is also created to provide blood flow to the pulmonary arteries (PAs) and an atrial septectomy is performed to ensure mixing of oxygenated and deoxygenated blood between the atria [10,12]. In recent years, for premature or low-birth-weight infants, or those with life-threatening conditions immediately after birth, some centres have adopted a hybrid technique combining interventional catheterization and surgical procedures [13,14].

The second stage, the Glenn procedure or bidirectional cavo-pulmonary connection (BDCPC), is the creation of a connection between the superior vena cava (SVC) and the PAs to reduce the volume load on the systemic ventricle (SV) by directing systemic venous return from the upper body to the lungs [15,16,17]. This stage improves diastolic function, reduces the ventricular wall thickness, and improves the function of the atrioventricular (AV) valve, all of which contribute to improving ventricular function [16,17].

The final interventional step is the total cavo-pulmonary connection (TCPC) operation, which completes the separation of pulmonary and systemic by allowing return of blood from the systemic to the pulmonary vascular bed, connecting the inferior vena cava (IVC) to the PAs [18,19,20]. The original Fontan procedure involved creating a connection pathway between the IVC and PAs to allow systemic venous blood to flow passively into the lungs; in this way, the workload of the SV is reduced. The TCPC, a refinement of the Fontan procedure, employs an extracardiac tunnel to connect the IVC and PAs directly. This approach enhances venous flow to the lungs, while minimizing complications such as turbulence and ventricular dysfunction. Some studies have shown that the creation of fenestration can reduce morbidity following the TCPC procedure [21]. Fenestration has been shown to decrease the incidence of pleural effusions and reduce the likelihood of developing protein-losing enteropathy and other late complications associated with TCPC hemodynamic.

Patients with HLHS face significant risks of circulatory failure from birth, with the greatest risk of death occurring in the first months of life [8,22,23]. Heart transplantation is the only long-term solution for end-stage circulatory failure, but it is complicated by comorbidities and poor outcomes, particularly in patients with severe end-organ dysfunction [6]. The pre-transplant condition greatly influences survival, and while ventricular assist devices can improve pre-transplant conditions, they are rarely used in HLHS infants [23,24]. Additionally, the development of anti-human leukocyte antigen antibodies can lead to complications such as antibody-mediated rejection and early graft failure [25,26]. Overall post-transplant survival varies by the stage of palliation, with infants transplanted after the Norwood operation facing the highest risk of death [24]. Advances in treatment of circulatory failure have improved outcomes in this population, making heart transplantation a crucial component of care for patients with HLHS [27,28].

In patients with HLHS, echocardiography is the imaging modality of choice; however, cardiac magnetic resonance (CMR) imaging and cardiac computed tomography (CCTA) play important roles at different stages of the patient’s clinical pathway [28,29,30,31,32,33,34,35,36,37,38,39,40,41,42,43,44,45,46]. The combined use of these techniques, depending on the stage and the specific needs of the patient, provides more precise management and optimal surgical planning.

This review emphasizes the crucial role of multimodality imaging, including echocardiography, CCTA, and CMR, in the diagnosis, monitoring, and treatment planning of HLHS (Table 1).

## 2. Multimodal Assessment During Fetal Life in Hypoplastic Left Heart Syndrome

Fetal echocardiography is a cornerstone of CHD screening, typically performed between the 18th and 22nd weeks of gestation [47]. The standard fetal cardiac anomaly scan includes imaging of the four-chamber view, outflow tract views, and the three-vessel tracheal view (3VTV). Suspicion of HLHS arises when the four-chamber view reveals severe asymmetry between the ventricles, LV hypoplasia, and structural abnormalities of the left-sided valves. The 3VTV complements this by enabling the early detection of major vessel abnormalities, such as hypoplasia or atresia of the ascending aorta and aortic arch, which may be identified as early as the first trimester [48]. These findings allow for early diagnosis and the scheduling of serial assessments every 4–6 weeks.

Once the diagnosis of HLHS is confirmed, detailed anatomical and physiological assessments are essential. These should evaluate systemic and pulmonary arterial and venous connections, ventricular function, TV regurgitation, reversed flow in the ascending aorta, and ASDs with shunt direction to determine the severity of HLHS and plan perinatal interventions [49]. In particular, pulmonary venous assessment should confirm—on colour Doppler—the drainage of at least one right and one left pulmonary vein into the posterior left atrium and include pulsed-wave Doppler sampling 1–2 mm proximal to the veno-atrial junction. Normal tracings display predominant systolic and diastolic forward flow with only minimal reduction during atrial contraction, whereas marked pulsatility with reduced/absent diastolic forward flow, prominent atrial reversal, or a to-and-fro pattern suggests an intact/restrictive atrial septum and the potential need for urgent postnatal atrial decompression.

A prenatal diagnosis is critical for ensuring delivery in a tertiary centre capable of providing immediate specialized care, including intravenous prostaglandin E1 (PGE1) therapy, advanced cardiac monitoring, and timely surgical or catheter-based interventions. High-risk features identified during fetal follow-ups can guide delivery planning and postnatal management. Notable prenatal warning signs include an intact atrial septum (IAS) or restrictive foramen ovale (r-PFO), RV dysfunction, mitral stenosis, aortic atresia, and abnormal ventricle-to-coronary artery communications [50]. For fetuses with IAS or r-PFO, fetal balloon septoplasty can be considered to enlarge the atrial communication, potentially improving both postnatal and long-term outcomes [51,52].

For selected cases of critical aortic stenosis (AS), fetal balloon valvuloplasty has emerged as an innovative therapy. By improving antegrade flow through the aortic valve, this procedure may promote prenatal LV development and support the possibility of a two-ventricle circulation [22]. However, more data is needed to establish its superiority over traditional approaches, given its procedural complexity and associated risks.

**Figure 1:**
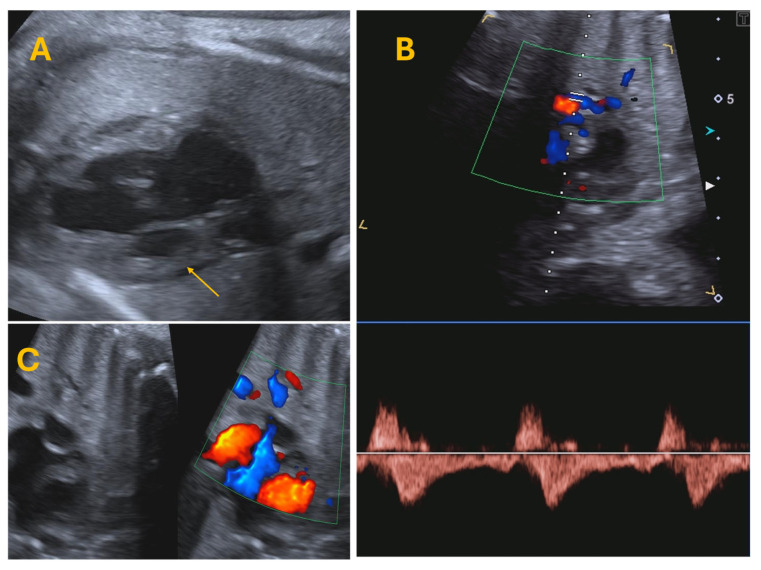
(**A**) four chamber view showing an example of Hypoplastic Left Heart Syndrome (arrow). (**B**) PV PW Doppler analysis, showing a reversed A wave. (**C**) PFO investigation, showing a thick PFO with left-right shunt.

Fetal CMR is an emerging tool that addresses several limitations of fetal echocardiography in CHD diagnosis. It provides detailed evaluation of the interatrial septum and left-to-right atrial shunts, making it instrumental in the diagnosis of IAS or r-PFO and in planning for delivery at specialized centres [53]. When echocardiographic windows are suboptimal, fetal CMR can also help clarify pulmonary venous anatomy and exclude anomalous connections, supporting risk stratification and perinatal planning. Additionally, fetal CMR enables the detection of the “nutmeg lung pattern,” indicative of pulmonary lymphangiectasia, which is associated with poor neonatal prognosis and low postnatal survival rates (Figure 1).

Advanced fetal CMR techniques, such as myocardial strain analysis using feature tracking (FT), allow for assessment of fetal RV radial and longitudinal function. This technique provides crucial insights into ventricular deformation but remains limited by the high fetal heart rate and spontaneous fetal movements that can compromise image quality [54,55].

## 3. Multimodal Assessment During Neonatal and Pediatric Life Until TCPC Completion

**Figure 2:**
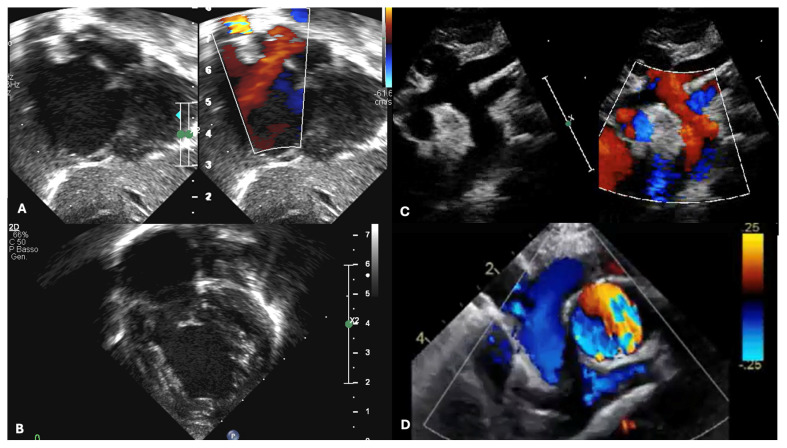
TTE evaluation focused on different cardiac structures that are subject to changes in the various phases of palliation. (**A**) Subcostal view demonstrating a PFO with a left-to-right shunt of a neonate with HLHS. (**B**) An apical 4-chamber view of a neonate with HLHS. (**C**) aortic arch in HLHS (**D**) Imaging of the superior cavo-pulmonary anastomosis from a suprasternal frontal view in a patient with Glenn: the SVC has been cut and connected to the right PA.

The diagnosis of HLHS is most often made prenatally. However, early postnatal diagnosis through echocardiographic evaluation can still occur in the first days of life. The post-delivery period is critical as the neonate transitions from fetal to extrauterine circulation, with significant changes such as decreasing pulmonary vascular resistance (PVR) and the closure of fetal shunts, including the PDA and PFO (Figure 2). One of the first echocardiographic projections required postnatally focuses on visualizing the PDA. The PDA typically appears wide and straight, with low-velocity flow (<2 m/s) directed right-to-left. Maintaining a patent PDA is essential for systemic perfusion, necessitating the initiation of prostaglandin E1 (PGE1) therapy. Infants with a prenatal or perinatal diagnosis of an IAS or r-PFO may require immediate intervention with balloon atrial septoplasty [52,56]. This is the reason why echocardiographic evaluation of the atrial septum must be performed promptly together with PDA evaluation.

Detailed echocardiographic assessment of left heart structures is vital to confirm the HLHS diagnosis and identify specific subtypes. For instance, forms of HLHS associated with aortic or mitral stenosis have been linked to poorer outcomes, including increased mortality and transplantation rates, as reported by Wilson et al. [6,57,58]. Additionally, anomalies in ventricular-to-coronary artery connections, while rare, are more frequently observed in cases with stenotic valve phenotypes and increase morality rate [58].

Assessment of the ascending aorta, aortic arch, and isthmus dimensions is crucial to evaluate cerebral perfusion. An aortic diameter <2 mm is often associated with cerebral hypoperfusion and increased postoperative risk [59]. Doppler imaging from the suprasternal notch can sample antegrade diastolic flow in the PDA and MPA and retrograde systolic flow in the aortic arch, indicative of retrograde cerebral and coronary perfusion.

Echocardiographic imaging, using apical four-chamber and parasternal views, typically reveals a dilated and hypertrophic RV and a hypoplastic, non-apex-forming LV. As the RV assumes the role of systemic circulation, assessing its function is critical. Integrating multiple functional methods ensures a comprehensive evaluation. Simple measurements such as tricuspid annular plane systolic excursion (TAPSE) and fractional area change (FAC) are reproducible and valuable for longitudinal follow-up, although FAC is limited by low reproducibility [60,61,62,63]. Emerging techniques like 3D echocardiography enable precise evaluation of RV volumes and ejection fraction, while speckle-tracking offers validated assessments of RV myocardial deformation, enhancing the understanding of single ventricle performance [64,65,66]. In healthy children, reference values include an average RV global longitudinal strain (2D-STE) of about −29% (with RV free-wall values slightly more negative), and 3D RV volumes should be reported as sex- and BSA-indexed z-scores from pediatric nomograms; normal 3D RV ejection fraction typically lies in the mid-50s to low-60s [67].

RV dysfunction and tricuspid valve incompetence are significant predictors of mortality across the stages of palliation [34,68,69].

**Figure 3:**
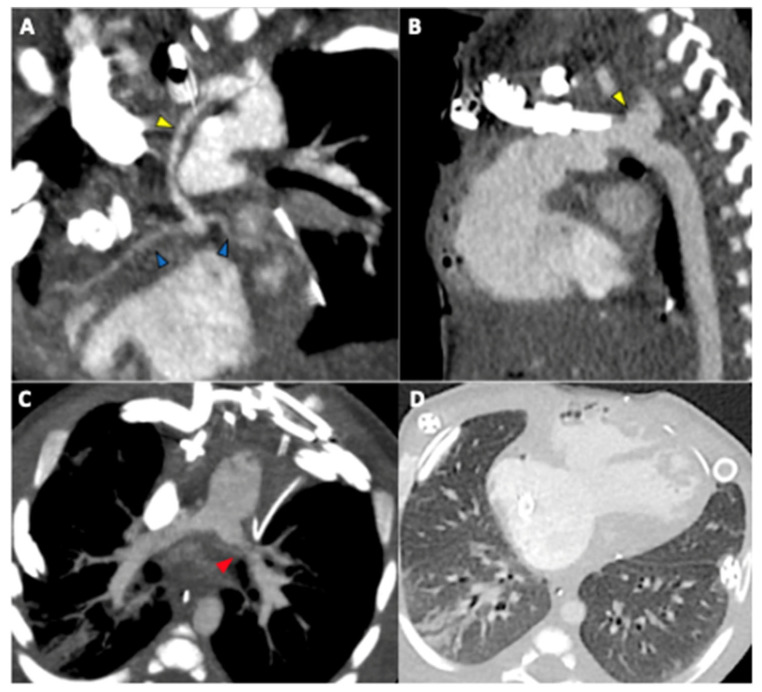
A fourteen days-old newborn with HLHS, scanned in sedation in extracorporeal membrane oxygenation (ECMO) cardio-pulmonary support at 130 bpm, s/p first stage of surgical palliation. In subfigure (**A**), the hypoplastic ascendant aorta (yellow arrowhead) and the coronaries (blue arrowheads). In subfigure (**B**), the descending aorta (yellow arrowhead). In subfigure (**C**), the stenosis of left PA (red arrowhead), and in subfigure (**D**), a right lung consolidation.

In select cases, CMR may serve as a preoperative imaging modality, particularly in specialized centres. CMR provides detailed anatomical and functional information in patients with complex anatomy, such as aortic arch or pulmonary vein anomalies. However, its use in neonates requires general anesthesia due to the prolonged imaging times and the need for patient immobility. Alternatively, CCTA can be employed before staged palliation when echocardiographic findings are inconclusive or raise concerns about complex anatomy. CCTA’s shorter imaging duration and wider availability make it a practical alternative, as it often does not require general anesthesia (Figure 3). CCTA is particularly helpful in identifying associated anomalies such as anomalous pulmonary venous drainage, interrupted aortic arch, and abnormalities of the systemic veins. The enhanced visualization of coronary arteries and aorto-pulmonary collaterals (APCs) provided by CCTA is another important information for these patients [41,43,70]. It also offers critical insights into the lungs, particularly in assessing conditions like lymphangiectasia, as well as airways, where tracheobronchial anomalies are more common in HLHS patients [6,41,42,71,72].

**Figure 4:**
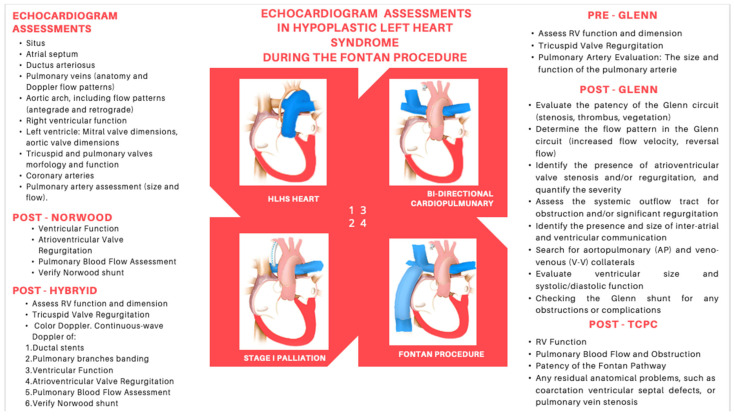
Optimal flowchart of the echocardiogram assessment of a child with HLHS before and during the three-step Fontan/TCPC completion.

Surgical planning for HLHS typically involves a series of accurate evaluations. The echocardiography enables the preoperative assessments, the intraoperative monitoring, which is often carried out with a transoesophageal or epicardial technique, and the post-surgery evaluation, useful for detecting any complications such as ventricular dysfunction, valve regurgitation, or shunt obstruction, which may require further surgical management (Figure 4) [8,10,70,72,73,74,75,76].

Following the Norwood procedure, it is necessary to assess the atrial septectomy using the subcostal view. The septectomy is often incomplete, with residual atrial tissue frequently detectable. Inadequate resection of the atrial septum can lead to postoperative cyanosis. In the case of a Blalock–Taussig shunt, which connects the right subclavian artery to the PA, the patency of the conduit can be assessed using Doppler from the suprasternal notch view. A patent conduit typically shows a sawtooth pattern on continuous Doppler; increased turbulence and velocity should raise suspicion of conduit stenosis. The Sano shunt directly connects the RV to the PA and is more challenging to visualize. Typically, the insertion points, narrowing along its course, and flow variations in the PAs are evaluated by the Doppler. The optimal windows to assess the Sano shunt are the subcostal view or the modified apical view, tilting the transducer to obtain a very anterior window. Additionally, the integrity of the neo-aortic valve, ascending neo-aorta, and aortic arch must be evaluated. Doppler assessment of these structures allows for the detection of stenosis or regurgitation of the valve or obstructions of the neo-aorta or arch [77].

In selected cases, it is possible to assess the success of the procedure using CMR and CCTA. In CMR, angiographic sequences allow for the evaluation of the integrity of the neo-aorta after reconstruction. The assessment of shunts (such as Blalock–Taussig or Sano type) is performed through dark blood imaging sequences or it is also possible after angiography sequences. Three-dimensional whole-heart sequences and appropriable phased angiographies can rival the presence of venous–venous or aorto-pulmonary collaterals. Phase contrast sequences are essential for calculating the Pulmonary-to-systemic flow ratio Qp/Qs ratio and also to quantify the hemodynamic effect of possible collaterals. The evaluation of atrial septal resection and ventricular function is also feasible and reliable.

CCTA is effective in tracking serial changes in both ventricular volumes and function, PA size, the size of hypoplastic ascending aorta, and the presence of ventricular septal defect and coronary ventricular communication [70]. In some cases, with HLHS palliated with Sano modification of the Norwood procedure, complex cardiovascular anatomy derived from CCTA datasets has been used for computational fluid dynamics [70]. CCTA is also helpful in identifying rare complications, such as pseudoaneurysm of the RV following the Sano shunt [78].

To perform the BDCPC, the second stage of palliation that occurs between 3 and 6 months, it is necessary to identify systemic venous connections, the size of the pulmonary arteries, and a non-restrictive flow of the interatrial septum before surgery [79]. Pulmonary artery adequacy can be gauged by the Nakata index (sum of branch pulmonary artery cross-sectional areas/BSA) and the McGoon ratio; in practice, Nakata ≥150–200 mm^2^/m^2^ (≥200 ideal) and McGoon ≥1.5 are commonly accepted benchmarks for BDCPC, provided PVR is low and there is no significant branch stenosis or distortion. Measurements should be taken just proximal to first lobar branching, avoiding focal stenoses. After the BDCPC procedure, the anastomosis between the SVC and the PA is visualized through the suprasternal notch view; at the anastomosis level, Doppler characteristics are a laminar flow, low velocity, and a biphasic pattern. Obstructions may be identified in the case of increased velocity and gradients >3 mmHg.

In CMR, the assessment of the anastomosis between the SVC and the PA can be performed using dark blood, cine, or gadolinium sequences. Generally, flows are low velocity, and the Qp/Qs calculation uses flow in the SVC and flow in the branches of the pulmonary arteries.

Following BDCPC, CCTA provides useful information in evaluating the relationship between the branch PAs and the airways, especially when considering interventions like stenting of the left PA, which could compromise the left main bronchus [42].

In a recent study, both CCTA and CMR imaging post-BDCPC showed significant clinical impact in over 40% of cases, with no significant difference in the impact of findings between the two modalities [73].

The TCPC procedure is performed between the ages of 2 and 4 and completes the palliation. Before and after the procedure, a complete echocardiographic evaluation is necessary, including the study of the interatrial septum, RV, TV, aorta, pulmonary arteries, and veins. For Fontan candidacy, many centres target Nakata ≥200–250 mm^2^/m^2^ and McGoon ≥1.8, acknowledging that acceptable outcomes may still be achievable with slightly lower values when hemodynamics and anatomy are favourable (low PVR and unobstructed, symmetric branch pulmonary arteries). The Fontan pathway, which connects the IVC to the PA, can be visualized through subcostal and apical windows. Identifying any obstruction in the conduit is critical and can be detected through colour flow assessment or by evaluating variations in velocity and gradients on Doppler.

A comprehensive evaluation of right ventricular function and tricuspid regurgitation must be precisely performed after each stage of palliation. Right ventricular dysfunction or severe tricuspid regurgitation can cause immediate hemodynamic compromise in the postoperative period. Recently, multiple studies have shown that speckle-tracking echocardiography is useful in assessing right ventricular function during progressive palliation while RV volume estimation using the 3D technique produces promising results [62,64].

However, further research is needed to develop optimal protocols for evaluating RV function in HLHS.

The CMR allows to identify the presence of any obstructions or clots in the entire systemic venous pathway.

After TCPC, CCTA should be considered to obtain further information on Fontan hemodynamic, or to assess small collaterals, coronary arteries, and lungs. As patients age, acoustic windows tend to diminish in quality, making CCTA an increasingly important modality for evaluating branch PAs, the IVC connection, and the tunnel [6].

Postoperative complications such as stenosis, thrombosis, or residual shunts are effectively evaluated with CCTA, which also plays a crucial role in assessing the patency of surgical conduits and the morphology of PAs and veins [70].

Pediatric patients are particularly vulnerable to the long-term risks of radiation due to their smaller body sizes and longer life expectancy, which increases their cumulative exposure over time [6,42]. Although low-dose radiation protocols have been adopted to mitigate this risk, the concern remains, particularly since HLHS patients often require repeated imaging throughout their lives to monitor their cardiovascular status [6,70]. Therefore, careful consideration is necessary when choosing CCTA over other imaging modalities, especially in the context of routine follow-up [6,71,73,80].

## 4. Multimodal Assessment During Pediatric and Adult Life Post-TCPC Completion

Post-TCPC echocardiography represents the first method to comprehensively evaluate patients with HLHS, allowing cardiac function and hemodynamic balance assessment, and early identification of potential complications [35]. Given the complex physiological adaptations of TCPC hemodynamic, rigorous echocardiographic surveillance is imperative for optimizing patient outcomes and preventing adverse events. In routine practice, this should include systematic assessment of cavo-pulmonary pathways, fenestration patency, and Doppler-derived indices of venous return to anticipate decompensation.

Right ventricular function through TAPSE and FAC must be assessed at every follow-up and correlate well with exercise performance in patients with HLHS after TCPC [81]. The AV ratio of systolic to diastolic duration (S/D) is assessed by measuring systolic and diastolic duration from the Doppler flow on tricuspid regurgitation. An increased S/D ratio is considered a geometric-independent Doppler method index of RV dysfunction as it results from a pathological shortened duration of diastole and elongation of the systole [82]. In adults with TCPC, AV S/D has been demonstrated to predict clinical outcomes and relate well with filling pressures obtained by cardiac catheterization [83,84]. Michel et al. assessed alterations in RV function after TCPC completion (1.6 to 5.1 years follow-up after surgery) by using both conventional and speckle-tracking echocardiography. They found FAC, E/A, E/e’ ratio, and global RV strain did not change during the follow-up period, while TAPSE and values of the systolic, early, and late diastolic RV strain rates dramatically dropped [65]. Speckle-tracking echocardiography has also been associated with outcomes in patients after TCPC. In a study by Ghelani et al., low circumferential ventricular strain presented the strongest association with mortality or need for heart transplant among all the echocardiographic variables evaluated [66].

Diastolic dysfunction is highly prevalent after TCPC and correlates with adverse clinical outcomes, especially in patients with longer follow-up [85]. In patients with single RV, invasive filling pressure has been demonstrated to correlate well with strain rate values, but not with tissue Doppler measurements, including the E/e’ ratio [69]. In HLHS cohorts after TCPC, typical systemic RV values are as follows: global longitudinal peak systolic strain rate ≈ −1.0 to −1.6 s^−1^, peak early diastolic strain rate ≈ 2.0–2.2 s^−1^, and peak late diastolic strain rate ≈ 0.9–1.2 s^−1^ (age/vendor/load dependent). However, the E:e’/end-diastolic volume ratio with a cutoff value of 0.26 mL^-1^ has demonstrated to reasonably identify diastolic dysfunction in pediatric patients after TCPC [86].

Additional insight into ventricular performance can be gained by the study of fluid dynamics [87,88]. Indeed, it has recently been shown that patients after TCPC exhibit elevated kinetic energy dissipation in conjunction with intense vortex flow rotation. At present, “normal values” for these fluid-dynamic indices are not standardized; Echo-PIV and blood speckle imaging provide modality- and vendor-dependent metrics (e.g., kinetic energy, energy-dissipation rate, vorticity/circulation, vortex-core strength) that are best interpreted when compared with age-matched biventricular controls or within-cohort centiles rather than absolute cut-offs [87,88].

AV valve regurgitation can develop and worsen at any time after TCPC, and when significant, it is associated with morbidity and mortality. Therefore, a careful evaluation of each patient is crucial. The mechanism can be various, from RV dysfunction to structural valvar apparatus abnormalities, to annular dilation [89].

Echocardiographic follow-up should include the examination of blood flow through the vena cava, Pas, and the assessment of any obstructions through the conduit. A low-velocity continuous flow with respiratory variation suggests unobstructed TCPCs.

A fenestration at the level of the ICV-PA conduit may be purposely left in situ to increase cardiac output. The fenestration flow can be studied by continuous wave Doppler: in the absence of obstruction, the mean gradient across the fenestration reflects the transpulmonary gradient. A gradient of 5 to 8 mmHg has been recognized in hemodynamically stable patients. If the fenestration has been closed with a device, it is valuable to visualize the device and any residual shunt [35].

The neo-aorta should be assessed as well. Cohen et al. described the dilation of the neo-aortic root following the TCPC operation, which can lead to the development of valvular regurgitation [90]. Common complications after aortic reconstruction in HLHS patients include anastomotic stenosis, aortic arch re-coarctation, aneurysms, neo-aortic root dilation, and neo-aortic valvular regurgitation, which can manifest even several years after the initial palliation stages [90,91]. Additionally, aortic deformations can have secondary effects on other structures, such as the single ventricle or the PAs. For instance, aortic dilation may lead to left PA compression, causing maldistribution of pulmonary blood flow, and subsequent impaired exercise capacity [92]. HLHS patients frequently exhibit altered bioelastic properties of the reconstructed aorta, causing higher systemic vascular resistance, and a subsequent afterload on the RV, ultimately leading to a decreased RVEF [93,94,95].

As patients age, acoustic windows tend to reduce in quality, making it difficult to gain from echocardiography all the fundamental information leading to the implementation of CMR and CCTA methods. Indeed, CMR is considered the gold standard for non-invasive evaluation of RV systolic function in patients with HLHS [96]. Ballenberg et al. recently defined CMR reference values for this population after TCPC completion [96]. Indexed RV volumes have been shown to increase before RVEF deterioration, and correlate with adverse outcomes, hence representing a reliable functional marker [97,98,99,100]. An indexed end-diastolic volume of the single ventricle >125 mL/m^2^ has been identified as a predictor of death or heart transplant [101]. CMR with catheterization may provide additional value by assessing load-independent parameters and intrinsic RV contractility in HLHS patients, finding a correlation between reduced exercise capacity and decreased preload during stress [102]. In addition, evolving CMR techniques—such as motion-compensated free-breathing 3D whole-heart acquisitions, real-time/accelerated cine imaging, and strain quantification by feature-tracking—enhance surveillance when breath-holding is limited and improve reproducibility of serial measurements [40,100,103,104].

Early signs of a failing TCPC circuit may also be detected through ventricular strain analysis, both global and regional, through CMR-FT. Global circumferential (GCS) and longitudinal (GLS) strains deteriorate earlier than EF and are associated with adverse outcomes, including death, the need for a transplant, protein-losing enteropathy, and plastic bronchitis [40,100,103,104]. In contemporary CMR-FT series of TCPC patients with a systemic RV (predominantly HLHS), representative values were as follows: GCS ≈ −20% to −22% (mean ~−21%) and GLS ≈ −16% to −18%. Systemic RVS showed less negative GCS than systemic LVS, while GLS is similar, and both tend to worsen on serial follow-up [40,100,103,104]. Regional strain analysis could identify areas of myocardial damage in case of previous Sano shunt, or reduced myocardial perfusion associated with previous Blalock–Taussig shunt, as well as assessment of intraventricular desynchrony. The latter is more common in single RV than in single LV, especially in patients with larger LV remnants, and is associated with reduced ventricular function, death or heart transplantation [105,106]. Myocardial tissue characterization (LGE and parametric mapping) complements functional indices, helping to phenotype myocardial disease burden over time [95]. CMR has a paramount role in assessing APC, a common complication in HLHS patients, particularly in case of late age at TCPC palliation [107,108,109,110]. APC can be calculated through phase-contrast sequences, as follows:

(1) $$APC=(Q_{Right\;pulmonary\;veins}+Q_{Left\;pulmonary\;veins})\;-(Q_{Right\;PA}+Q_{Left\;PA})$$

Or:

(2) $$APC=Q_{Ascending\;aorta}-(Q_{Superior\;vena\;cava}+Q_{Descending\;aorta})$$

The first equation should be preferred in patients with a previous Norwood procedure, where the accuracy for phase-contrast flow velocity in the ascending aorta may be undermined by competing flows at the aorto-pulmonary anastomosis and turbulent flow in the large reconstructed aortic arch [106]. Detection and quantification of APC are particularly important, as they are correlated with central venous pressure, Brain natriuretic peptide (BNP), history of hemoptysis, and NYHA functional class. Notably, if compared to other TCPC groups, HLHS patients exhibit a higher risk for volume overload from APC flow [111].

Beyond morphology and flows, 4D flow CMR quantifies kinetic energy, viscous energy loss, and vorticity; adverse energetics are linked to impaired exercise capacity and liver fibrosis and may localize to the TCPC tunnel and LPA, highlighting potential targets for optimization [112,113,114]. Computational fluid dynamics can further support multidisciplinary planning by simulating virtual conduit expansion and its hemodynamic impact under rest and stress conditions [115].

**Figure 5:**
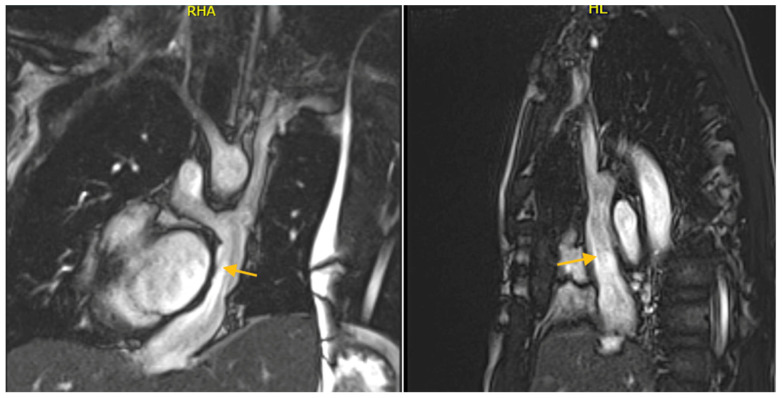
Coronal and sagittal view of a CMR in a twenty-year-old patient with a previous bilateral bidirectional Glenn procedure and TCPC completion. The yellow arrows indicate the external conduit.

CMR is essential for evaluating conduits that can develop stenoses, either relative or absolute, or thrombi (Figure 5). The inferior cava connection and tunnel should be evaluated for phasic flow and checked for thrombotic formations using early gadolinium sequences. Studies have revealed CMR incidental findings of clinically silent thrombi, even not detected by echocardiography, suggesting the importance of routine multi-modality imaging in this population [116].

Given the cumulative radiation exposure connected to CCTA, this should be reserved for scenarios in which it adds clear value over CMR [6,70]. CCTA is particularly useful for monitoring structural changes over time in HLHS patients, including the assessment of the TCPC circuit, PAs, coronary arteries, and extracardiac structure [70,71]. However, in centres with advanced CMR expertise, motion-corrected whole-heart imaging and targeted flow assessments will often obviate the need for CT in routine surveillance, with CT retained as a problem-solving tool [6,70].

Postoperative complications such as stenosis, thrombosis, or residual shunts are effectively evaluated with CCTA [70]. These findings are critical, as they directly impact patient management, potentially leading to re-intervention, catheter-based interventions, or surgery [70]. One particularly concerning issue in adults with TCPC circuit is the development of venous and pulmonary thrombosis. CCTA excels in detecting these complications, providing detailed imaging that can guide anticoagulation therapy or decisions about surgical intervention. In addition, atrial thrombi, which are more common in older patients who have undergone an atrio-pulmonary TCPC, can be visualized accurately with CCTA, allowing for timely management [6,69]. Another area where CCTA plays a crucial role is in the detection of venovenous collaterals and aorto-pulmonary collaterals, which may contribute to cyanosis or heart failure. These small vessels are often difficult to detect with other imaging techniques but can be visualized with high clarity using CCTA, enabling early intervention to prevent worsening symptoms [71]. CCTA enables the identification of lymphatic abnormalities, pulmonary parenchymal changes, and bronchial obstructions.

## 5. Conclusions

Different imaging modalities play an important role in the diagnosis, prognosis, and follow-up of patients with HLHS (Table 2). Echocardiography remains the first-line method for prenatal diagnosis and subsequent evaluation during the various stages of palliation. It is the most widely used modality among centres due to its low cost, wide availability, and absence of radiation. Echocardiographic markers also carry prognostic value: prenatal intact/restrictive atrial septum is consistently associated with higher early mortality, and in post-Fontan patients systemic RV strain (GLS/GCS) independently predicts transplant-free survival, often outperforming conventional indices [117].

Conversely, CMR is extremely important in the evaluation of patients with a single ventricle, as it is the gold standard for assessing volumes and right ventricular function and for evaluating mitral valve regurgitation; such information is closely related to the prognosis of patients with HLHS. Additionally, several studies have demonstrated its utility in fetal assessment of ventricular function. Furthermore, CMR provides critical insights into the patency of shunts and systemic circulation. CMR-derived ventricular size and function are strong predictors of outcomes after the Fontan operation—indexed end-diastolic volume (EDVi) and systemic RV ejection fraction show independent associations with transplant-free survival—and, on fetal MRI, the ‘nutmeg lung’ pattern is linked to markedly increased early mortality [101].

Although CCTA is limited by radiation exposure, it offers exceptional spatial resolution, making it crucial for the comprehensive visualization of the TCPC circuit and for excluding thrombi or collaterals in the circuit. One of the key strengths of CCTA is its role in the long-term follow-up of patients and the visualization of extracardiac structures for the early diagnosis of post-palliation complications. Importantly, a greater burden of aorto-pulmonary collaterals is associated with poorer transplant-free survival, underscoring the value of CT (and CMR) in detecting and quantifying these lesions during surveillance [118].

Before and after the various stages of palliation, a thorough evaluation of HLHS patients should involve the complementary use of echocardiography, CMR, and CCTA. The selection of imaging modality should consider the centre’s expertise and the patient’s specific characteristics to effectively guide clinical and surgical management and define the prognosis of patients diagnosed with HLHS. Beyond hard outcomes, people with a Fontan circulation report lower health-related quality of life than healthy peers; imaging-derived markers of ventricular size and deformation parallel functional limitation and can help identify patients at risk for impaired wellbeing.

## Figures and Tables

**Table 1:** Advantages and disadvantages of different imaging modalities.

Echocardiography	CMR	CCTA
Advantages	Advantages	Advantages
Low cost Widely available Non-invasive Radiation-free Prenatal diagnosis with the planning of delivery and early interventions	Radiation-free Non-invasive, can be performed without contrast Alternative to echocardiography in prenatal diagnosis for complex cases	High spatial resolution Rapid acquisition Non-invasive
Disadvantages	Disadvantages	Disadvantages
Limited availability (specialized training/ specialized centre) Limited image quality in poor acoustic windows Operator dependent	Limited availability (specialized/research centres for CHD/fetal CMR) Fetal CMR still considered research method, additive to echo Contraindicated in patients with non-conditional devices Longer acquisition time and cooperation (breath-holding) required Gadolinium contraindicated in pregnancy (it should be used only if absolutely essential)	Requires radiation exposure ~0.3–1.5 mSv Potential reaction to iodinate contrast agents Requires cooperation Reduced temporal resolution Contraindicated in prenatal period Requires use of iodinated contrast medium

CMR: cardiac magnetic resonance, CTTA: cardiac computational tomography angiography, CHD: congenital heart disease.

**Table 2:** Multimodality imaging pathway for Hypoplastic Left Heart Syndrome.

Life Phases	Stage of Palliation	Echocardiography	Magnetic Resonance Imaging	Cardiac Computed Tomography Angiography
Fetal life	Prenatal diagnosis	Structure and function of the heart chambers; Assessments of valvular diseases; Assessments of the systemic and pulmonary arterial and venous connections; Prenatal warning signs: IAS or r-FO.	Structure and function of the heart chambers; Assessments of valvular diseases; Assessments of the systemic and pulmonary arterial and venous connections; Prenatal warning signs: IAS or r-FO; detection of nutmeg lung pattern.	**/**
Neonatal and Pediatric life	Initial assessment pre-Norwood/Sano	Structure and function of the heart chambers; Assessments of valvular diseases; Evaluations of the DA and atrial septum; Assessments of the systemic and pulmonary arterial and venous connections; Anomalous connections of the coronary arteries; Cerebral blood flow and celiac trunk flow.	For selected cases (e.g., arch or pulmonary vein anomalies): Define the patient’s anatomical structures; Assessments of single ventricle function; Assessments of valvular diseases; Evaluations of the DA and atrial septum.	For selected cases (e.g., arch or pulmonary vein anomalies): Define the patient’s anatomical structures; Assessment of any residual cardiac problems (e.g., anomalous pulmonary venous drainage, interrupted aortic arch, and ventricular septal defects); Assessment of any residual systemic problems (e.g., tracheobronchial anomalies).
	Post-Norwood/Sano	RV function and dimension; TV regurgitation; PA function and dimension; Assessment of pulmonary blood flow; Assessment of atrial septectomy; Evaluation of Norwood or Sano shunt.	If concerns not addressed by echocardiography (e.g., shunt, PAs, arch, Ao-PA connection): RV function and dimension; TV regurgitation; Assessment of atrial septectomy; Evaluation of the neo-aorta integrity; Evaluation of Norwood or Sano shunt.	If concerns not addressed by echocardiography (e.g., shunt, PAs, arch, Ao-PA connection): Assessment of PA size; Evaluation of the neo-aorta size; Assessment of complex cardiovascular anatomy; Evaluation of Norwood or Sano shunt; Identifying rare complications (e.g., pseudoaneurysm of the RV).
	Post-Glenn	RV function and dimension; Assessment of AV valve stenosis and/or regurgitation; Assessment of outflow tract; Evaluation of Gleen circuit patency.	If concerns not addressed by echocardiography (e.g., shunt, PAs, arch, Ao-PA connection): RV function and dimension; TV regurgitation; Assessment of outflow tract; Assessment of pulmonary blood flow; Demonstration of venovenous and aorto-pulmonary collaterals; Evaluation of Gleen circuit patency.	If concerns not addressed by echocardiography (e.g., shunt, PAs, arch, Ao-PA connection): Assessment of relationship between the branch pulmonary arteries and the airway; Demonstration of venovenous and aorto-pulmonary collaterals; Evaluation of Gleen circuit patency.
	Post-TCPC	RV function and dimension; TV regurgitation; Assessment of pulmonary blood flow; Evaluation of circuit patency; Assessment of any residual anatomic problems (e.g., coartaction, ventricular septal defects, or pulmonary vein stenosis).	If concerns not addressed by echocardiography: RV function and dimension; Assessment of pulmonary blood flow (e.g., APC); Evaluation of circuit patency; Identifying complications (e.g., obstructions or clots); Assessment of systemic problems.	If concerns not addressed by echocardiography: Assessment of morphology of PAs and veins; Assessment of aorto-pulmonary collaterals; Evaluation of circuit patency; Identifying complications (e.g., stenosis, thrombosis, or residual shunts); Assessment of systemic problems.
Adult life	Follow-up post-TCPC	RV function and dimension; TV regurgitation; IVC dimension; Assessment of pulmonary blood flow; Evaluation of circuit patency; assessment of the neo-aorta size and complication; Assessment of any residual anatomic problems (e.g., coartaction, ventricular septal defects, or pulmonary vein stenosis).	If concerns not addressed by echocardiography: RV function and dimension; Assessment of pulmonary blood flow (e.g., APC); Evaluation of circuit patency; Identifying complications (e.g., obstructions or clots); Assessment of systemic problems (e.g., lymphatic imaging).	If concerns not addressed by echocardiography: Monitoring structural changes; Assessment of morphology of PAs and veins; Assessment of coronary arteries anomalies; Evaluation of circuit patency; Identifying complications (e.g., stenosis, thrombosis, or residual shunts); Assessment of systemic problems; Evaluation of extracardiac structure.

## Data Availability

Not applicable.

## Abbreviations

3VTV	Three-Vessel and Trachea View
ASD	Atrial Septal Defect
AV	Atrioventricular
APC	Aorto-Pulmonary Collaterals
S/D	Atrioventricular Ratio of Systolic to Diastolic Duration
BDCPC	Bidirectional Cavo-Pulmonary Connection
BNP	Brain Natriuretic Peptide
CHD	Congenital Heart Disease
CMR	Cardiac Magnetic Resonance
CCTA	Cardiac Computed Tomography Angiography
EF	Ejection Fraction
FAC	Fractional Area Change
FO	Foramen Ovale
FT	Feature Tracking
GCSs	Global Circumferential Strains
GLSs	Global Longitudinal Strains
HLHS	Hypoplastic Left Heart Syndrome
IAS	Intact Atrial Septum
ICV	Inferior Vena Cava
LGE	Late Gadolinium Enhancement
LPA	Left Pulmonary Artery
LV	Left Ventricle
NYHA	New York Heart Association
MPA	Main Pulmonary Artery
PAs	Pulmonary Arteries
PDA	Patent Ductus Arteriosus
PGE1	Prostaglandin E1
PV	Pulmonary Vein
PVR	Pulmonary Vascular Resistance
PWD	Pulsed-Wave Doppler
r-PFO	Restrictive Foramen Ovale
RV	Right Ventricle
RVEF	Right Ventricle Ejection Fraction
SV	Systemic Ventricle
SVC	Superior Vena Cava
TAPSE	Tricuspid Annular Plane Systolic Excursion
TCPC	Total Cavo-pulmonary Connection
TTE	Transthoracic Echocardiography
TOE	Transesophageal Echocardiography
TV	Tricuspid Valve
